# Occurrence of Antibiotics, Antibiotic Resistance Genes and Viral Genomes in Wastewater Effluents and Their Treatment by a Pilot Scale Nanofiltration Unit

**DOI:** 10.3390/membranes11010009

**Published:** 2020-12-23

**Authors:** Maria Beatriz Cristóvão, Solomon Tela, Andreia Filipa Silva, Micaela Oliveira, Andreia Bento-Silva, Maria Rosário Bronze, Maria Teresa Barreto Crespo, João Goulão Crespo, Mónica Nunes, Vanessa Jorge Pereira

**Affiliations:** 1 iBET, Instituto de Biologia Experimental e Tecnológica, Apartado 12, 2781-901 Oeiras, Portugal; b.cristovao@ibet.pt (M.B.C.); solomon.tela@ibet.pt (S.T.); andreiasilva@ibet.pt (A.F.S.); micaelaoliveira@ibet.pt (M.O.); mrbronze@ff.ulisboa.pt (M.R.B.); tcrespo@ibet.pt (M.T.B.C.); mnunes@ibet.pt (M.N.); 2 LAQV-REQUIMTE, Department of Chemistry, Faculdade de Ciências e Tecnologia, Universidade Nova de Lisboa, 2829-516 Caparica, Portugal; jgc@fct.unl.pt; 3 Instituto de Tecnologia Química e Biológica António Xavier, Universidade Nova de Lisboa, Av. da República, 2780-157 Oeiras, Portugal; 4 Faculdade de Farmácia, Universidade de Lisboa, 1649-003 Lisboa, Portugal; abentosilva@ff.ulisboa.pt

**Keywords:** antibiotics, antibiotic resistance genes, viral genomes, wastewater effluents, occurrence, pilot-scale treatment, nanofiltration

## Abstract

Broad-spectrum fluoroquinolone antibiotics (ciprofloxacin and levofloxacin), carbapenem and fluoroquinolone resistance genes, as well as viral genomes, were detected in grab samples of wastewater effluents. Passive samplers, which are simpler and easier to use and provide information about the concentrations and combination of contaminants present in a certain fluid matrix over time, proved to be extremely promising devices to monitor the presence of the target antibiotics in wastewater effluents. Nanofiltration was tested with a pilot-scale unit installed at a domestic wastewater treatment facility, using a Desal 5DK membrane operated at a constant transmembrane pressure of 6 bar and 70% recovery rate. In a 24 h experimental assay, the variation of the membrane permeance was low (6.3%). High rejections of the target contaminants from the wastewater effluent were obtained by the pilot-scale treatment. Hence, nanofiltration using the Desal 5DK membrane is considered to be a promising treatment to cope with chemical and biological contaminants present in wastewater effluents.

## 1. Introduction

Population growth, urbanization, industrialization, agricultural expansion and climate change globally intensified massive freshwater consumption [1]. This, in turn, increases wastewater production, which, if not effectively treated, can pose a pollution risk to the ecosystem and human health due to the presence of contaminants (e.g., pharmaceutically active compounds and pathogenic microorganisms such as antibiotic-resistant bacteria and viruses) in the discharged effluents.

Regarding pharmaceutically active compounds, special interest should be given to antibiotics since this group of drugs is widely consumed [2], persists in wastewater and drinking water treatment [3], and facilitates the development of antibiotic-resistant bacteria and antibiotic resistance genes, which can cause deleterious human health effects [4,5].

According to a recent report from the United Nations interagency coordinating group on antimicrobial resistance, at least 700,000 people die each year due to drug-resistant diseases [2]. The same report estimates that these diseases could force up to 24 million people into extreme poverty by 2030, cause 10 million deaths per year by 2050 and damage the economy as devastatingly as the 2008–2009 global financial crisis [2]. Although treating many diseases in animals and humans relies on the use of effective antibiotics, it is extremely urgent to control their use.

A growing number of studies have focused on the occurrence of antibiotics in the aquatic environment, and several antibiotics have been detected in hospital effluents, wastewater effluents and surface waters [3,4]. Particular attention should be given to broad-spectrum antibiotics like quinolones and carbapenems that have a high potential for resistance development [5]. Following this, according to the European Commission implementing decision 2018/840, some antibiotics such as ciprofloxacin (a fluoroquinolone) have been included in the watch list of substances for Union-wide monitoring. This decision is in alignment with the European one health action plan against antimicrobial resistance, which encourages the use of this watch list to improve the worldwide knowledge of antimicrobials occurrence in the environment [6]. Predictions based on structures and physicochemical properties indicated that ciprofloxacin and levofloxacin are expected to be present in wastewater effluents to a greater extent compared to carbapenems due to their low biodegradability [7]. Loos et al. analyzed 156 polar organic chemical contaminants (including a large number of antibiotics) in effluents from 90 European wastewater treatment plants (WWTPs) and found out that ciprofloxacin was among the most frequently detected contaminants, with a frequency of detection around 90% [8]. Some studies have also already reported the occurrence of antibiotic-resistant bacteria and genes in wastewater effluents and in the receiving water bodies [9,10].

Additionally, other pathogenic agents, like viruses, can also persist in raw wastewater and treated wastewater as well as in the receiving water bodies [11]. One of the main sources of viral pathogens in wastewater is the human fecal matter from infected persons [12,13,14] that can shed 10^5^ to 10^12^ viral particles per gram of fecal matter [15]. Besides human pathogenic viruses, waterborne viruses that originate from food production, animal husbandry, seasonal surface runoff and other sources are also present in wastewater [16]. The abundance and diversity of pathogenic viruses in wastewater have been shown to reflect the pattern of infection in the human population [11,17]. Adenovirus (AdV), rotavirus (RoV), hepatitis A virus (HAV), and other enteric viruses, such as noroviruses (NoV), coxsackievirus, echovirus, reovirus and astrovirus, are some of the principal human pathogens viruses transmissible via water media.

Hence, there is an urgent need for the development of effective treatment solutions as an alternative to the conventional wastewater treatment processes to avoid the release of these contaminants in the aquatic environment. In this way, membrane filtration processes such as nanofiltration and reverse osmosis could be considered a promising solution for the treatment of effluents with several emerging contaminants like antibiotics, antibiotic resistance genes and viruses. Nanofiltration membranes may be used to produce high-quality wastewater effluents in a more sustainable way than reverse osmosis membranes due to their lower energy consumption and higher throughput. Indeed, laboratory-scale studies conducted on the removal of antibiotics, antibiotic resistance genes, and viruses (both enveloped and non-enveloped) based on nanofiltration technology have demonstrated promising results (e.g., [18,19,20]).

This work focused on testing the occurrence of the broad-spectrum fluoroquinolone antibiotics ciprofloxacin and levofloxacin in real wastewater effluents. Grab samples were collected during different sampling dates throughout a year. The use of passive samplers was also evaluated for the detection of these two compounds by determining a time-weighted average concentration and comparing it with the results obtained from the grab samples. Additionally, antibiotic resistance genes and pathogenic viral genomes were also quantified in the same wastewater effluent. Finally, the removal of these contaminants was addressed using a nanofiltration pilot-scale unit installed in the same sampling site of the WWTP.

## 2. Materials and Methods

### 2.1. Occurrence Study

In this study, the occurrence of the target antibiotics, antibiotic resistance genes, as well as viral genomes, were analyzed in wastewater samples. All the wastewater samples were collected after the biological treatment and prior to the disinfection step at a wastewater treatment utility. The average pH value of the wastewater samples collected during a year was 7.1 ± 0.1. For each target contaminant, different protocols were followed, which are detailed on the following sections.

#### 2.1.1. Antibiotics

Regarding the antibiotics, ciprofloxacin and levofloxacin were selected as target drugs in this study. Both target drugs are broad-spectrum antibiotics with different structures, and their physicochemical properties are represented in Table 1.

##### Sampling Campaign

Since antibiotics are expected to be present in the aquatic environment at trace concentration levels (often µg·L^−1^ or ng·L^−1^), solid-phase extraction is commonly used to preconcentrate the samples. However, these conventional procedures are usually costly and time-consuming. As an alternative, the use of effective passive samplers that provide real information about the concentrations of different contaminants and their combinations present in different matrices over time was also tested since these samplers may constitute a much more accurate approach than relying on intermittent grab samples that may misrepresent average concentrations due to short-term temporal variability. In this study, the two sampling approaches (grab and passive sampling) were, therefore, performed for the detection of the target antibiotics.

In the first sampling approach, wastewater effluent samples were collected on different dates during a year (from October 2018 until December 2019). These samples were stored in glass bottles, transported to the laboratory and kept at 4 °C under dark conditions. All wastewater effluent samples were filtered with 1.2 µm filters (GE Healthcare, Amersham, UK) and 0.45 µm polyamide membrane filters (Filter-Lab, Barcelona, Spain) to avoid the clogging of the solid phase extraction cartridges used during the concentration step.

In addition, the potential use of polar organic chemical integrative samplers (POCIS) for the detection of ciprofloxacin and levofloxacin was assessed. For this, a canister containing duplicate POCIS was deployed in the same sampling site previously described, with a rope at two m-depth, during three different days of a week (from 9 a.m. until 6 p.m.). In parallel, grab wastewater samples were collected on the same days in the morning and afternoon periods. After their collection, all POCIS were kept in an aluminum foil bag, transported to the laboratory and kept at −20 °C until extraction. Moreover, two POCIS were used as a field blank, and none of the target contaminants were detected on their extracts.

##### Solid Phase Extraction Procedures

All grab samples were subjected to a sample concentration procedure using a stationary phase (Oasis HLB cartridges 200 mg, six cc; Waters, Milford, MA, USA) previously optimized for the detection of anticancer drugs [21]. Briefly, 500 mL of sample were loaded into the cartridge at a flow rate of 5 mL·min^−1^ under vacuum, after which the cartridge was rinsed with 3 mL of laboratory grade water and then dried for approximately one hour. Methanol (Carlo Erba reagents, Barcelona, Spain) was then used to elute the compounds from the cartridge. Finally, the extracted sample was concentrated with a gentle nitrogen stream until a final volume of 500 µL was achieved. All samples were stored at −20 °C, filtered and were further analyzed by UPLC-MS/MS. Following this procedure, the percent recovery of each target antibiotic spiked in the same wastewater effluent was 21 ± 5% and 49 ± 1%, for ciprofloxacin and levofloxacin, respectively. These recovery values were considered to correct the measured concentrations of the target antibiotics in the wastewater effluent.

Regarding the passive samplers, Pharmaceutical–POCIS (ExposMeter, AB, Tavelsjö, Sweden) that contain 200 mg of OASIS HLB sorbent comprised between two microporous polyethersulfone membranes were used. The extraction procedure consisted of simpler and less time-consuming steps. First, each POCIS was clean with laboratory-grade water, and then the sorbent was gently transferred into an empty 6 mL SPE cartridge and placed between two polyethylene frits (Supelco, Bellefonte, PA, USA). After, 6 mL of methanol was used to elute the target compounds from the cartridges and the obtained extract was dried with a gentle stream of nitrogen until a final volume of 500 µL was achieved. All the samples were stored at −20 °C until further analysis.

After the UPLC analysis, the mass of analyte accumulated in each POCIS was determined and, since it was assumed that POCIS were deployed during their linear uptake regime, Equation (1) was used to estimate the time-weighted average concentrations:

(1) $$M_{s}=R_{s}C_{\mathrm{TWA}}t$$

where M_s_ is the amount of analyte accumulated in the sorbent (ng), R_s_ is the sampling rate (L·day^−1^), C_TWA_ (ng·L^−1^) is the time-weighted average concentration, and t is the time of deployment (days).

For the determination of the time-weighted average concentrations, the sampling rate, i.e., the volume of water cleared per unit of time for a given compound, must be estimated. In this study, the sampling rate obtained by Bailly et al. for ofloxacin (an isomer of levofloxacin) was assumed to be the same for ciprofloxacin and levofloxacin (0.1 L·day^−1^) [22]. This sampling rate has already been used by Ory et al. to estimate the concentration of ciprofloxacin in an hospital effluent [23].

##### Analysis by Liquid Chromatography with Tandem Mass Spectrometry

The analyses were performed on a Waters^®^ Acquity™ ultra-high-performance LC. The separation was performed after injection of a 10 µL sample on a reversed-phase column (HPLC/UPLC Mediterranean Sea 18; 2.2 μm 100 × 2.1 mm) at 35 °C. The mobile phase consisted of 0.1% (*v*/*v*) formic acid in Milli-Q water (A) and 0.1% (*v*/*v*) formic acid in acetonitrile (B) with a flow rate of 0.30 mL·min^−1^. Tandem mass spectrometry (MS/MS) detection was performed on an Acquity™ triple quadrupole (Waters^®^, Dublin, Ireland) using an electrospray ionization source operating at 130 °C and applying a capillary voltage of 2.2 kV. The compounds were ionized in positive ion mode (ESI+). Analytical conditions and collision energies were optimized for each compound. All analyses were performed in multiple reaction monitoring (MRM) mode in order to achieve a higher selectivity and sensitivity. Two transitions were used in order to identify and quantify the antibiotics in the different samples. The MS/MS conditions optimized for each target antibiotic are presented in Table 2 and the gradient conditions optimized for the separation of the compounds are depicted in Table 3. High purity nitrogen (N_2_) was used both as drying gas and as a nebulizing gas. Ultra-high purity argon (Ar) was used as collision gas. MassLynx software (version 4.1; Waters^®^, Dublin, Ireland) was used to control the system, for data acquisition and processing. 

Before analysis by UPLC-MS/MS all samples as well as the calibration standards were filtered with 0.2 µm filters (GE Healthcare, Amersham, UK).

Selectivity, linearity (range 5 to 500 µg·L^−1^), precision and accuracy of the analytical method were determined for the target antibiotics, ciprofloxacin (Sigma Aldrich, Saint Louis, MO, USA) and levofloxacin (Sigma Aldrich, Saint Louis, MO, USA). The results obtained are presented in the Supporting Information section and discussed as proposed in the EU Commission Decision 2002/657/EC according to the following criteria: (i) the retention time of the compound in the real sample must not vary more than ±2.5% from the retention times corresponding to the calibration standards, (ii) the MRM1/MRM2 ratio for target compounds in samples do not deviate more than 20–30% when comparing with the same values obtained for the calibration standards, and (iii) the signal/noise ratio must be higher than 10.

#### 2.1.2. Antibiotic Resistance Genes

##### DNA Extraction

The water samples were primarily filtered in triplicate through 0.45 µm pore-size polyethersulfone filters (Pall Corporation, New York, NY, USA) and the resulting filtrates were again filtered through 0.22 µm pore-size polyethersulfone filters (Pall Corporation, New York, NY, USA). Filtration volumes were defined by clogging of the filters as a measure of the same amount of retained biomass. After filtration, the DNA was extracted from each of the two filters per sample following the standard protocol from the DNeasy PowerWater Kit (Qiagen, Hilden, Germany), being recovered in 50 µL elution buffer. At the end, the DNA extracted from both related filters was mixed. DNA concentrations and purity were measured using a NanoDrop 1000 spectrophotometer (Thermo Fisher Scientific, Waltham, MA, USA) and stored at 4 °C for further use.

##### Detection and Quantification of the Target Resistance Genes by TaqMan Multiplex qPCR

According to their importance in terms of clinical relevance and global distribution, five carbapenem-*bla_KPC_*, *bla_OXA-48_*, *bla_NDM_*, *bla_IMP_* and *bla_VIM_*- and three (fluoro)quinolone- *qnrA*, *qnrB* and *qnrS*- resistance genes were chosen to be detected and quantified by three TaqMan multiplex qPCR assays previously developed and optimized [24].

The quantification of the target carbapenem (*bla*) and (fluoro)quinolone (*qnr*) resistance genes, as well as of the 16S rRNA gene, was conducted in triplicate on a LightCycler 96 real-time PCR system (Roche, Basel, Switzerland) using the following program: DNA denaturation/polymerase activation at 95 °C for 5 min; 40 cycles of amplification at 95 °C for 10 s and 60 °C for 30 s. Information about the mix reactions of each TaqMan qPCR was detailed by Oliveira et al. [24].

#### 2.1.3. Viruses

##### Concentration of Viral Particles from the Water Samples

Viral particles concentration was made by organic flocculation with skimmed milk, based on a procedure previously described [25]. Flocculants were allowed to sediment for 8 h, and centrifuged at 8000× *g* for 40 min. The pelleted viral concentrate was suspended in 8 mL phosphate buffer (1:2 (*v*/*v*) mixture of 0.2 M Na_2_HPO_4_ and 0.2 M NaH_2_PO_4_), and the viral concentrate was stored at −80 °C until viral nucleic acids extraction.

##### Viral DNA and RNA Extraction and cDNA Preparation

Nucleic acids were extracted from 140 µL-portions of the respective viral concentrate using the QIAamp Viral RNA minikit (Qiagen, Valencia, CA, USA), following the manufacturer’s instructions. Nucleic acids were dissolved in 40 µL of RNase free elution buffer (AVE), and the concentration and purity of the obtained extracts determined using a NanoDrop 1000 spectrophotometer. Total RNA was converted to cDNA in a final volume of 20 µL with the NZY First-Strand cDNA synthesis kit (NZYTech, Lisboa, Portugal) according to the manufacturer’s instructions. The nucleic acids were stored at −80 °C until further use.

##### Detection and Quantification of the Viral Genomes by TaqMan Multiplex qPCR

Multiplex qPCRs protocols for the simultaneous detection of viral genomes of HAV, NoV GI and GII and HEV (MqPCR 1) and adenovirus and polyomavirus (MqPCR2) were previously developed and optimized (data not showed). The MqPCR 1 reaction was carried out in a total volume of 20 μL using SensiFAST™ Probe No-ROX amplification mix (Bioline, London, UK). The concentrations of HAV, NoV GI and GII, and HEV for forward and reverse primers were 100 nM and 400 nM, respectively, and for the probes were 100 nM for NoV GII, HAV and HEV, and 250 nM for HEV. Regarding the MqPCR2 the concentrations of the forward and reverse primers were 300 nM and for each probe (AdV, PyV) were 100 nM. The MqPCR 1 temperature profile was: 5 min at 95 °C as hot start, and 40 cycles of 15 s at 95 °C for denaturation, 1 min at 60 °C for annealing, and 1 min at 65 °C for extension. For the MqPCR2 the temperature profile was: 5 min at 95 °C as hot start, and 40 cycles of 10 s at 95 °C for denaturation and 30 s at 60 °C for annealing. Thermal cycling, fluorescent data collection, and data analysis were performed in a LightCycler 96 real-time PCR system (Roche), according to the manufacturer’s instructions.

### 2.2. Nanofiltration Experimental Assay

The efficiency of a nanofiltration system for the retention of the target antibiotics, antibiotic resistance genes as well as viral genomes was assessed by conducting an experimental assay in the same sampling site where the occurrence samples were collected. A nanofiltration pilot unit was placed after the biological treatment and, consequently, the viability of using nanofiltration as tertiary treatment in a wastewater treatment plant was assessed.

A submersed pump was constantly collecting the effluent from the biological treatment into a 1 m^3^ tank, which was then connected to the pilot unit. The pilot unit comprised one feed pump, one pressurization pump as well as one recirculation pump, which ensured a recirculation of 900 L·h^−1^. Additionally, two pre-filters (70 µm and 30 µm) were located at the entrance of the pilot unit to protect the membrane. The membrane used consisted of a spiral wound Desal 5DK module (model DK4040F30, Suez membranes, Lenntech, Delfgauw, The Netherlands), with an active membrane surface area of 7.9 m^2^. The Desal 5DK membrane is a thin film composite membrane with polysulfone support layer and is negatively charged at neutral pH. Prior to the experimental assay, the permeance of the membrane was measured with tap water and the obtained value was 3.5 L·h^−1^·m^−2^·bar^−1^ (20 °C).

The operation of the pilot unit has been optimized in a previous study conducted with the same effluent and several assays were conducted under controlled permeate flux (12.7 L·m^−2^·h^−1^, 15.2·L·m^−2^·h^−1^, 19.0 L·m^−2^·h^−1^ and 25.3 L·m^−2^·h^−1^) or controlled transmembrane pressure conditions (5 bar and 6 bar) and different recovery rates (feed flow of water converted into treated flow of water (permeate); approximately 20%, 30%, 40%, 70% and 80%) to determine the operating conditions that would minimize fouling resistance, would maximize the production of treated water and rejection of anticancer compounds [26]. The best operating conditions for this wastewater effluent were found at a controlled pressure difference of 6 bar and using a recovery rate of approximately 70%. Hence, a 24 h experimental assay was performed using the same conditions.

All samples from feed, permeate and retentate were analyzed for the target antibiotics, antibiotic resistance genes and viral genomes, following the protocols described in the above section.

The apparent rejection of each target contaminant was calculated using Equation (2):

(2) $$\mathrm{Rejection}\;\left(\%\right)=\left(1-\frac{C_{P}}{C_{f}}\right)\times 100$$

where C_p_ is the concentration of the target contaminant in the permeate side and C_f_ is the concentration of the target contaminant in the feed.

## 3. Results and Discussion

### 3.1. Occurrence of the Target Contaminants

#### 3.1.1. Antibiotics

**Figure 1:**
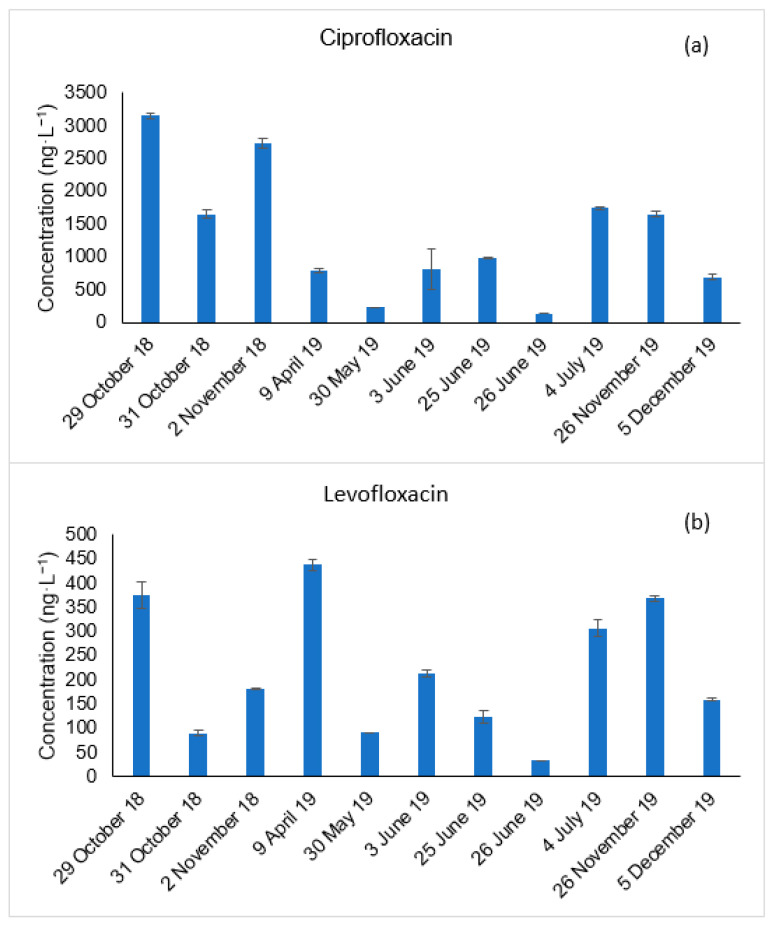
Occurrence of ciprofloxacin (**a**) and levofloxacin (**b**) in a wastewater effluent at different periods of a year.

The occurrence of ciprofloxacin and levofloxacin in the wastewater effluent was evaluated from October 2018 until December 2019. Figure 1 represents the concentration of the target antibiotics obtained from grab wastewater effluent samples.

As represented in Figure 1, both target antibiotics were detected in all the collected samples and their concentration did not vary much between the different seasons of the year, which could be expected since these broad spectrum antibiotics are used to treat a wide variety of bacterial infections.

Regarding ciprofloxacin (Figure 1a), its concentration ranged between 135 and 3150 ng·L^−1^. The lowest measured concentration was on 26 June 2019, whereas the highest concentration was obtained on 29 October 2018. Similar occurrence values have been reported in the literature. For example, Verlicchi et al. detected ciprofloxacin in several wastewater effluent samples collected from a WWTP located in the Northern of Italy with average concentrations of 630 ng·L^−1^ [27]. In addition, Rossmann et al. detected ciprofloxacin at 920 ng·L^−1^ in a wastewater effluent from a WWTP located in Germany [28].

In the same way, levofloxacin (Figure 1b) was detected in all the wastewater samples in concentrations ranging from 34 ng·L^−1^ to 438 ng·L^−1^. The minimum and maximum concentrations were recorded on 26 June 2019 and 9 April 2019, respectively. These values are also in agreement with others already reported in the literature. For example, levofloxacin has been detected in wastewater effluents of two WWTPs located in Slovakia at concentrations up to 58 ng·L^−1^. In addition, Rossmann et al. detected levofloxacin in concentrations up to 836 ng·L^−1^ [28].

**Figure 2:**
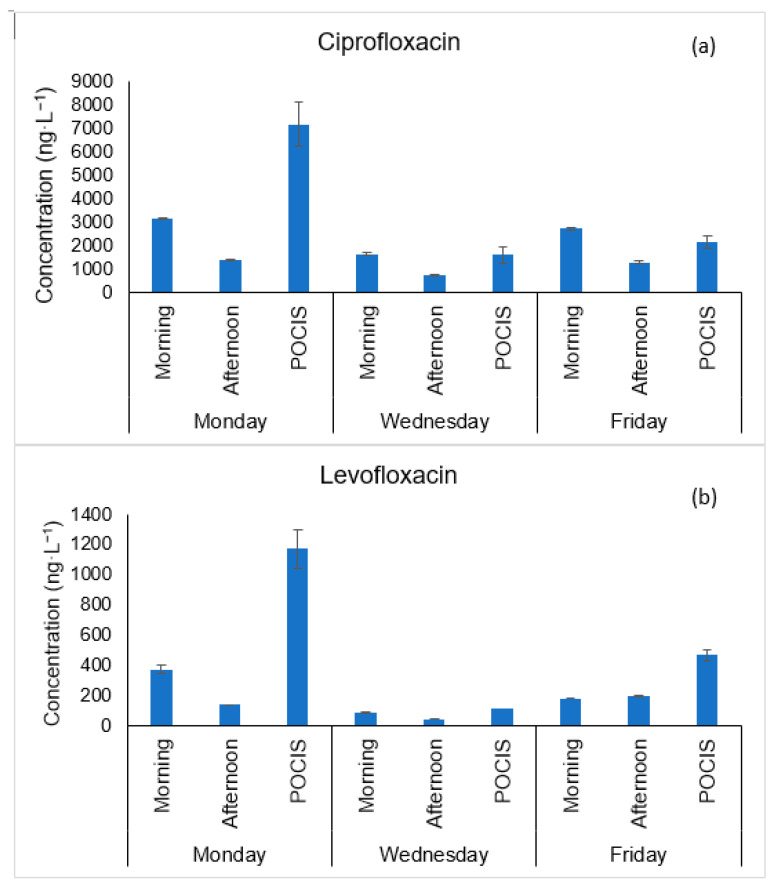
Concentration of ciprofloxacin (**a**) and levofloxacin (**b**) obtained from grab effluent samples collected on 29 October 2018 (Monday), 31 October 2018 (Wednesday) and 2 November 2018 (Friday) in the morning and afternoon period, as well as time-weighted average concentrations of each target compound obtained from polar organic chemical integrative samplers (POCIS), deployed on these days.

In addition, the potential use of the pharmaceutical-POCIS for the detection of the target antibiotics was assessed. For this, POCIS were deployed on the week of 29 October 2018–2 November 2018, on Monday, Wednesday and Friday from 9 a.m. until 6 p.m. The time-weighted average concentrations, estimated for each compound assuming a sampling rate of 0.1 L·day^−1^ and using Equation (1), are represented in Figure 2.

As represented in Figure 2, a good agreement was found between the time-weighted average concentrations obtained from the POCIS deployed on Wednesday and Friday with the concentrations obtained from the grab samples collected on these days in the morning and afternoon period. For example, on Wednesday, the estimated C_TWA_ value for ciprofloxacin was 1611 ng·L^−1^ and according to grab samples, on this day its concentration ranged from 752 to 1655 ng·L^−1^. In the same way, the C_TWA_ value estimated for levofloxacin was 118 ng·L^−1^ and the concentration estimated from the grab samples ranged from 45 to 90 ng·L^−1^. On the other hand, the results obtained on Monday for both antibiotics with the POCIS are much higher than the results obtained with the grab samples showing us that the grab sampling events may have missed higher occurrence levels of these two compounds in other periods of the day.

Future work should test the uptake profile of different chemical contaminants by exposing the samplers to wastewater effluents over several days so that more reliable sampling rates could be determined, since the calibration would be performed in situ.

Nevertheless, POCIS proved to be efficient in qualitatively and quantitatively monitoring the presence of ciprofloxacin and levofloxacin in wastewater effluents since both antibiotics were detected in all the POCIS extracts. For this reason, POCIS can be an useful tool for a first screening of the problem and it brings several advantages since it is simpler and less time-consuming when compared to the traditional grab samples.

Finally, the occurrence of both target antibiotics proves that, in some plants, conventional wastewater treatment may not guarantee their complete removal.

#### 3.1.2. Occurrence of Antibiotic Resistance Genes and Viral Genomes

**Figure 3:**
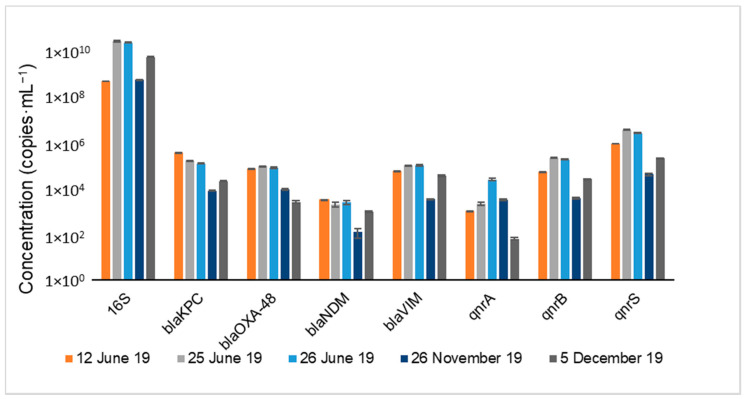
Occurrence of carbapenem (*bla_KPC_, bla_OXA-48_*, *bla_NDM_* and *bla_VIM_*) and (fluoro)quinolone (*qnrA, qnrB and qnrS*) resistance genes in wastewater effluent at different days. The data for *bla_IMP_* is not represented since it was lower than the detection limit in all the samples (1 copy·mL^−1^).

The occurrence of carbapenem (*bla_KPC_*, *bla_OXA-48_*, *bla_NDM_, bla_IMP_* and *bla_VIM_*) and (fluoro)quinolone (*qnrA*, *qnrB* and *qnrS*) resistance genes in the wastewater effluent at different days is represented in Figure 3. 16S rRNA gene is also represented in Figure 3 since its quantification was performed to assess the bacterial abundance on different days.

Regarding the studied antibiotic resistance genes, all the target carbapenem and fluoro(quinolone) resistance genes (*bla_KPC_*, *bla_OXA-48_*, *bla_NDM_*, *bla_VIM_*, *qnrA*, *qnrB* and *qnrS*) except *bla_IMP_* were detected in all the sampling days. The lowest concentration obtained was for the *qnrA* gene (59.5 copies·mL^−1^) on 5 December, and the highest concentration was for the *qnrS* (2.85 × 10^6^ copies·mL^−1^) gene on 25 June.

The high concentrations of carbapenem resistance genes present in the effluents reflect the increasing resistance in the microbial community towards this group of last-line antibiotics. Other studies recently published have reported the presence of carbapenem and (fluoro)quinolone resistance genes in the wastewater treatment processes of different European WWTPs [9,10,29], showing that most of these genes are present at higher concentrations in WWTPs of Southern Europe rather than in those of Northern Europe [29]. This is in line with the concentrations of carbapenem and (fluoro)quinolone resistance genes found in this study. This can be explained by the differences at the level of the antibiotic consumption pattern between Northern and Southern European countries, and possibly by the warmer waters of Southern Europe, which may be another favorable factor for the bacterial growth, including the bacteria-harboring resistance genes, being this a major driver of antibiotic resistance in the environment [29].

**Figure 4:**
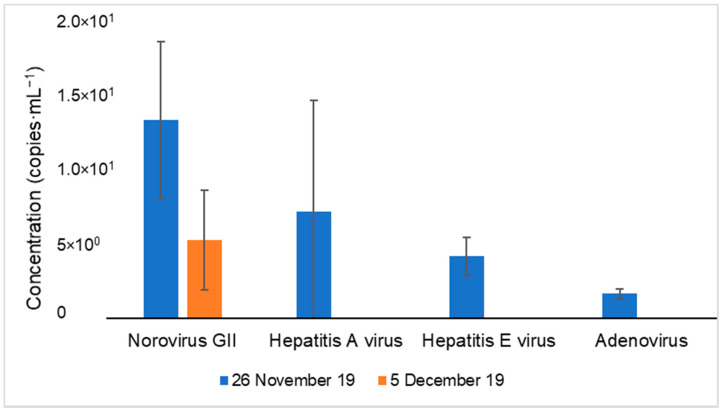
Occurrence of different types of viral genomes (norovirus GII, hepatitis A virus, hepatitis E virus and adenovirus) in wastewater effluent on two different days.

Regarding the target viral genomes, their detection was only possible on 26 November and 5 December 2019, and their concentration is represented in Figure 4.

Low concentration of the viral genomes NoV GII, HAV, HEV and adenovirus were detected, being the NoV GII genome the only one present on both days. Additionally, NoV GI and Polyomavirus genomes were not detected in any of the sampling days. The absence of these viral genomes in the collected samples does not necessarily mean that these genomes are not present in the effluents; it could be due to the detection limit of the multiplex qPCR protocols (1 copy of the genome·L^−1^).

Consistent results have also been reported by Teixeira et al. 2020 [28] in a WWTP where NoV GI and NoV GII genomes were detected in the effluent after the secondary treatment, with an average concentration of 10^5^ genome copies·L^−1^, and in the effluent after the tertiary treatment, with concentrations of 10^4^ genome copies·L^−1^. In addition, La Rosa et al. 2010, detected NoVGI/GII genomes in the influents and effluents of five WWTPs from Italy, with a higher prevalence of NoV GI, one of the most predominant type on the human population [30].

For HEV, Portugal is considered endemic for this virus [31,32], which is mainly present in pigs [33,34]. Thus, its release into the aquatic environment is expected, and consequently, its presence in the wastewater samples. Although in this study HEV genome was present at low concentrations, in another study from Matos et al. [34], the HEV genome was also detected but only in the influent samples of two Portuguese WWTPs. These authors did not indicate the HEV concentrations since their main goal was to perform molecular characterization of the virus. Similar results were obtained in a Spanish study [35], which detected HEV genomes only in the influent samples of four WWTPs, with average concentrations of 10^3^ genome copies·L^−1^. Additionally, a study from de-Beyer et al. revealed the presence of the HEV genomes in the effluent samples of several German WWTPs, with an average concentration of 10^3^ genome copies·L^−1^ [36].

### 3.2. Efficiency of Nanofiltration for the Removal of the Target Contaminants

The efficiency of a nanofiltration pilot-scale unit using the Desal 5DK membrane to remove the target antibiotics, antibiotic resistance genes and viral genomes from the wastewater effluent was evaluated.

Based on previously optimized conditions, the pilot unit was operated at a controlled pressure difference of 6 bar and an average recovery rate of 70% [26]. During the 24 h of in situ experiments, the permeance did not change much with an average normalized value (20 °C) of 2.8 L·h^−1^·m^−2^·bar^−1^ and a coefficient of variation of 6.3%. The membrane could therefore be operated using these conditions for significantly longer times before washing events are needed.

**Figure 5:**
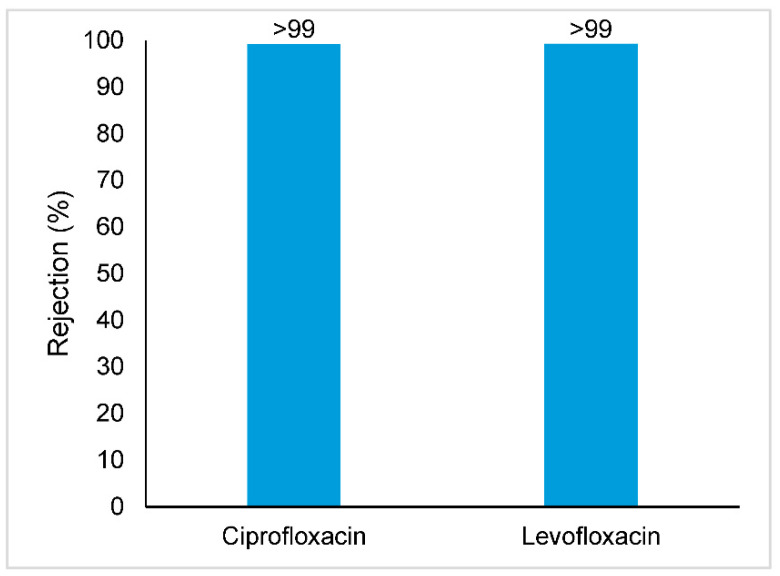
Removal of the target antibiotics from wastewater effluent using a Desal 5DK nanofiltration membrane; When the symbol “>” is used, it means that the compound was not detected in the permeate sample (below detection limit).

**Figure 6:**
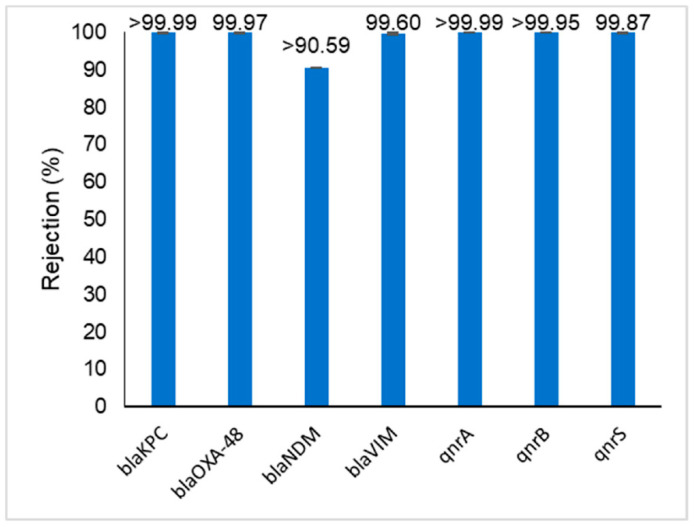
Removal of the target carbapenem (bla) and (fluoro)quinolone (qnr) resistance genes from wastewater effluent using a Desal 5DK membrane. When the symbol “>” is used, it means that this resistance gene was not detected in the permeate sample (below detection limit).

**Figure 7:**
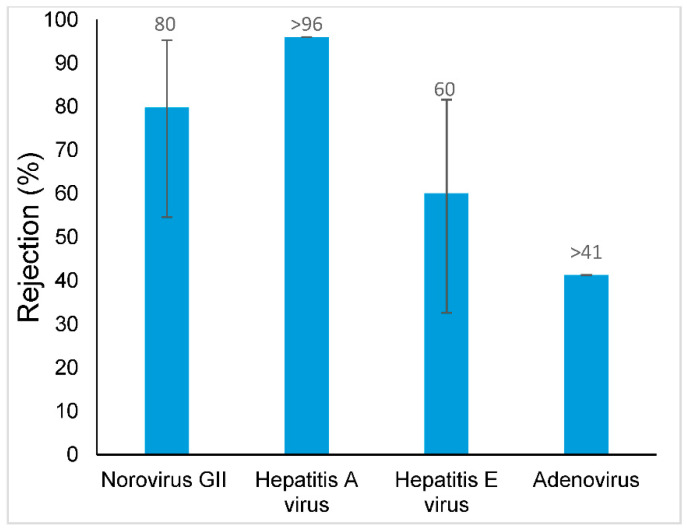
Removal of the target viral genomes from wastewater effluent using a Desal 5 DK membrane; When the symbol “>” is used, it means that this viral genome was not detected in the permeate sample (below detection limit).

The rejections obtained for the target antibiotics, antibiotic resistance genes and viral genomes are represented in Figure 5, Figure 6 and Figure 7, respectively.

As illustrated in Figure 5, high rejections were obtained for ciprofloxacin and levofloxacin. Both target antibiotics were not detected in the permeate samples. The percent rejection values were therefore calculated considering the lowest point measured in the calibration curve with a signal-to-noise ratio higher than 10. Taking into account the concentration factor and the recovery from the solid phase extraction procedure, that will correspond to a concentration of 24 ng·L^−1^ for ciprofloxacin and 10 ng·L^−1^ for levofloxacin in the permeate samples. It can be assured that the rejection was higher than 99% for both target compounds.

As it is well known, different mechanisms may be involved in the rejection of solutes by a nanofiltration membrane, such as size exclusion, electrostatic interactions and hydrophobic interactions. Based on the molecular weight and characteristics of both target antibiotics (Table 1) compared to the molecular weight cutoff of the membrane used, their rejection is expected to be mainly governed by size exclusion.

Dolar et al. reported rejections of ciprofloxacin in Milli-Q water higher than 99% when using an NF 270 and NF90 membrane, which are similar to the rejection values obtained with the Desal 5DK membrane used in this work, processing real wastewater after biological treatment [19].

As represented in Figure 6, the carbapenem resistance genes *bla_NDM_* and *bla_KPC_* as well the fluoroquinolones resistance genes *qnrA* and *qnrB,* were not detected in the permeate samples. The percent rejection values were therefore calculated considering the detection limit of 1 copy·mL^−1^. Regarding the other target resistance genes, even though high rejections were obtained, it was possible to detect them on the permeate samples, at levels ranging from 3.2 ± 3.1 copies·mL^−1^ (*bla_OXA-48_*) to 58.2 ± 3.5 copies·mL^−1^ (*qnrS*). Considering that this WWTP facility has the particularity of being in a closed environment, these low concentrations of some ARG could be due to the dissemination of aerosols near the sampling points of the pilot-scale unit.

The effective retention of the target carbapenem and fluoroquinolone resistance genes reported in this study agrees with results obtained by other authors. Slipko et al. showed that ultrafiltration, nanofiltration and reverse osmosis membranes could retain more than 99.8% of free DNA (pure plasmid and linear fragments of different sizes) [20]. Size exclusion was reported as the main retention mechanism. Lan et al. reported that nanofiltration and reverse osmosis treatment processes were extremely effective in retaining sulfonamide and tetracycline resistance genes (4.98–9.52 logs removal compared to raw sewage) [37]. The higher levels of rejection reported can be explained due to the extremely high levels of the sulfonamide and tetracycline resistance genes present in the raw sewage sampled by the authors (swine wastewater treatment). Lu et al. reported that a combination of microfiltration and reverse osmosis filtration processes could be used to effectively remove tetracycline, sulfonamide, macrolide and quinolone resistance genes from wastewater effluents [38]. Gros et al. studied the fate of pharmaceuticals and antibiotic resistance genes in a full-scale on-farm livestock waste treatment plant and reported that even though the reverse osmosis permeates had low levels of pharmaceuticals, antibiotic resistance genes copy numbers were still detected [39].

Figure 7 shows that the viral genomes from hepatitis A virus and Adenovirus genomes were not detected in the permeate samples, and consequently, their rejections were calculated assuming the detection limit of the method (1 genome copy·L^−1^).

Even though it was not expected that viral particles/viral fragments of the genome would pass through the nanofiltration membrane, Norovirus GII and hepatitis E viral genomes were detected in the permeate samples at levels lower than 4.3 genome copies·L^−1^. According to Van der Bruggen [40], viruses might not be completely removed by driven membrane processes due to the potential presence of “abnormally large” pores, which may lead to the leakage of viruses. Additionally, as mentioned above, the pilot-scale treatment process was operated in a real wastewater treatment plant where aeration facilities can generate and diffuse bioaerosols containing chemicals and microorganisms including viruses that are typically non-waterborne [41,42], being impossible to guarantee a sterile environment, and thus, contamination of the permeate samples cannot be ruled out.

It is important to note that the molecular techniques used in this study only provide estimates of the presence and abundance of the viral genomes in a sample, not measuring the viral infectivity. For a complete assessment of the presence of viruses in treated wastewater effluents, their presence, viability and potential infectivity should also be addressed by cell-line cultures studies.

Similar results were also obtained by Gimenez et al. 2009 where adenovirus and polyomavirus genomes were detected in some permeate samples collected in a drinking-water treatment plant after nanofiltration and reverse osmosis treatment. However, these samples did not show infectivity in the cell culture assays [39].

Overall, high removal of most target contaminants was achieved and thus, nanofiltration with a Desal 5DK membrane has the potential to be used as a tertiary treatment in a wastewater treatment facility and guarantee higher water quality. Nevertheless, long term assays should be conducted to study if adsorption of the target contaminants on the membrane is significant and guarantee there is no breakthrough of the compounds after several days of operation. Furthermore, if membrane filtration is applied, retentate treatment should be addressed, for example, by direct photolysis or advanced oxidation processes.

## 4. Conclusions

The fluoroquinolone antibiotics ciprofloxacin and levofloxacin, as well as several carbapenem and fluoroquinolone resistance genes, were detected in grab sampling events conducted throughout a year. When daily samples collected in grab events and using passive samplers were compared, the pharmaceutical polar organic chemical integrative samplers were found to be an extremely promising alternative to grab samples since their use avoids the most time-consuming steps of the solid phase extraction procedure. The occurrence of different types of viral genomes from Norovirus GII, hepatitis A virus, hepatitis E virus and adenovirus was also reported in one of the sampling events using the same wastewater effluent.

Nanofiltration using a Desal 5DK membrane was tested at pilot-scale to remove antibiotics, antibiotic resistance genes and viral genomes detected at occurrence levels in real wastewater effluent. The results of a 24 h assay conducted at a constant pressure of 6 bar showed that the permeance was maintained and that a high removal of antibiotics, antibiotic resistance genes and viral genomes can be expected with this treatment process. The use of nanofiltration as a tertiary treatment is, therefore, a promising solution to increase the quality of the wastewater effluents produced.

## Figures and Tables

**Table 1:** Main physicochemical properties of ciprofloxacin and levofloxacin.

Compound	Structure	Molecular Formula	Molecular Weight (Da)	Log K_ow_^a^
Ciprofloxacin	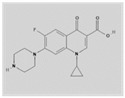	C_17_H_18_FN_3_O_3_	331.3	0.28
Levofloxacin	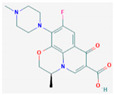	C_18_H_20_FN_3_O_4_	361.4	−0.39

^a^ Pubchem-NIH database-https://pubchem.ncbi.nlm.nih.gov/accessed August 2020.

**Table 2:** Optimization of tandem mass spectrometry (MS/MS) ion transitions and other parameters for detection of the target antibiotics.

Compound.	Ciprofloxacin	Levofloxacin
Retention time (min)	6.72	6.49
Precursor ion [M + H]^+^	332	362
Source potential (V)	50	50
Collision Energy (eV)	20	20
MRM1 transition	332 > 288	362 > 318
MRM2 transition	332 > 314	362 > 261

**Table 3:** Gradient conditions of mobile phases used in UPLC-MS/MS analysis.

Time (min)	0	1	7	8	8.10	10	10.10	20
% A	100	95	80	80	10	10	100	100
% B	0	5	20	20	90	90	0	0

A—0.1% (*v*/*v*) formic acid in Milli-Q water, B—0.1% (*v*/*v*) formic acid in acetonitrile.
